# The Roles of Endoplasmic Reticulum in NLRP3 Inflammasome Activation

**DOI:** 10.3390/cells9051219

**Published:** 2020-05-14

**Authors:** Yang Zhou, Zhizi Tong, Songhong Jiang, Wenyan Zheng, Jianjun Zhao, Xiangmei Zhou

**Affiliations:** 1 College of Animal Science, Southwest University, Chongqing 402460, China; Zhizi_tung@163.com (Z.T.); 15859788060@163.com (S.J.); keepmoving_zwy16@163.com (W.Z.); zhao8182@swu.edu.cn (J.Z.); 2 Immunology Research Center, Medical Research Institute, Southwest University, Chongqing 402460, China; 3 State Key Laboratories for Agrobiotechnology, Key Laboratory of Animal Epidemiology of the Ministry of Agriculture, National Animal Transmissible Spongiform Encephalopathy Laboratory, College of Veterinary Medicine, China Agricultural University, Beijing 100193, China; zhouxm@cau.edu.cn

**Keywords:** endoplasmic reticulum, NLRP3, inflammasome, IRE1α

## Abstract

The NLRP3 (nucleotide-binding domain, leucine-rich-repeat-containing family, pyrin domain-containing 3) inflammasome senses pathogen-associated molecular patterns (PAMPs) and danger-associated molecular patterns (DAMPs), and activates caspase-1, which provokes release of proinflammatory cytokines such as interleukin-1β (IL-1β) and IL-18 as well as pyroptosis to engage in innate immune defense. The endoplasmic reticulum (ER) is a large and dynamic endomembrane compartment, critical to cellular function of organelle networks. Recent studies have unveiled the pivotal roles of the ER in NLRP3 inflammasome activation. ER–mitochondria contact sites provide a location for NLRP3 activation, its association with ligands released from or residing in mitochondria, and rapid Ca^2+^ mobilization from ER stores to mitochondria. ER-stress signaling plays a critical role in NLRP3 inflammasome activation. Lipid perturbation and cholesterol trafficking to the ER activate the NLRP3 inflammasome. These findings emphasize the importance of the ER in initiation and regulation of the NLRP3 inflammasome.

## 1. Introduction

The innate immune system specifically senses pathogen-associated molecular patterns (PAMPs) such as lipopolysaccharide (LPS) [1], flagellin [2], peptidoglycan [3], double-stranded RNA (dsRNA) [4,5], and dsDNA [6], as well as danger-associated molecular patterns (DAMPs) such as DNA released from damaged mitochondria [7], extracellular ATP [8], amyloid-β [9], and monosodium urate [10], through a group of pattern recognition receptors (PRRs). Among these receptors, nucleotide-binding domain, leucine-rich-repeat-containing family, pyrin domain-containing 3 (NLRP3) is widely described. Organelles, including mitochondria, the ER and the Golgi, engage in NLRP3 inflammasome activation.

The ER, a dynamic and well-connected organelle, is involved in folding and transport of protein molecules along with lipid biosynthesis. The ER is highly sensitive to perturbation and plays a critical role in the function of many organelle networks [11]. During stress conditions, accumulation of misfolded and unfolded proteins within the ER triggers ER-stress response, which identifies a series of signals that deal with perturbations in ER homeostasis [12]. In this review, we discuss the roles of the ER in NLRP3 inflammasome assembly and ER-associated molecules in NLRP3 inflammasome activation.

## 2. Brief Introduction to the NLRP3 Inflammasome

The NLRP3 inflammasome is a multiprotein platform, which is composed of a sensor protein NLRP3, the adaptor apoptosis-associated speck-like protein containing a caspase-activation recruitment domain (ASC), and the cysteine protease caspase-1. NLRP3 consists of an N-terminal pyrin domain (PYD), a central nucleotide binding or oligomerization (NACHT) domain, and a C-terminal leucine-rich repeats (LRRs) motif which is associated with PAMP sensing and regulation of its activity [13]. Following NLRP3 activation, it interacts with ASC and caspase-1. The formation of macromolecular protein complex induces autocleavage and activation of caspase-1, which processes precursors of proinflammatory cytokines such as IL-1β and IL-18 to generate the active forms [14]. Caspase-1 activation also initiates programmed cell death named pyroptosis [15].

**Figure 1:**
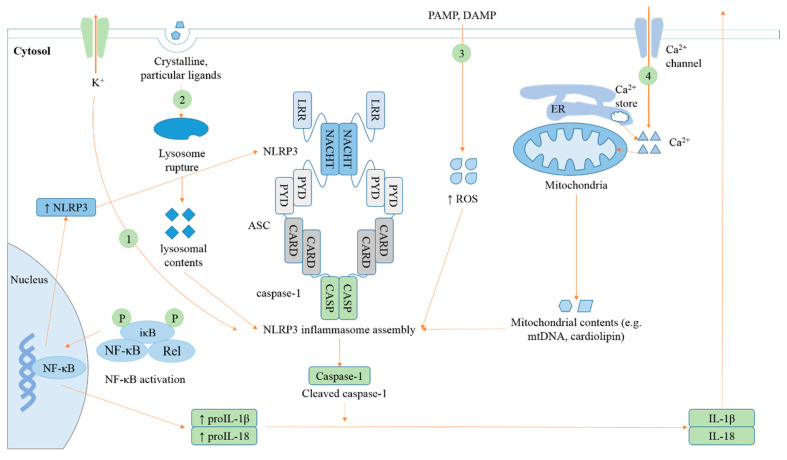
Models for nucleotide-binding domain, leucine-rich-repeat-containing family, pyrin domain-containing 3 (NLRP3) inflammasome activation. Four models for NLRP3 inflammasome activation that may not be exclusive have been proposed: (1) Multiple signal transduction pathways triggered by pathogen-associated molecular patterns (PAMPs)(/danger-associated molecular patterns DAMPs) converge on K^+^ efflux [20,21,22], leading to NLRP3–NEK (NIMA related kinase) interaction and NLRP3 inflammasome activation [23,24]. (2) Engulfment of crystalline or particular ligands, including monosodium urate (MSU) [25], silica [26], and amyloid-β [27], leads to lysosomal damage and resultant cytosolic release of lysosomal contents, which activates the NLRP3 inflammasome. (3) NLRP3 agonists, including ATP [28], MSU, and asbestos [29] trigger production of reactive oxygen species (ROS). This common pathway engages the NLRP3 inflammasome. However, later studies demonstrated that ROS only control NLRP3 inflammasome activation in the priming step, but not in the activation step [30]. ROS generation is even dispensable for both the priming and activation in certain circumstances [20]. (4) Ca^2+^ mobilization from extracellular milieu or endoplasmic reticulum (ER) Ca^2+^ stores induced by NLRP3 agonists leads to elevation of cytosolic Ca^2+^ concentration. Excessive and/or sustained mitochondrial Ca^2+^ influx results in Ca^2^^+^ overload, mitochondrial damage, and release of mitochondrial contents, triggering NLRP3 inflammasome activation [31,32,33].

The NLRP3 inflammasome is activated by a number of chemically- and structurally-unrelated stimuli [14]. Two signals are generally required in NLRP3 inflammasome activation: an NF-κB-dependent priming signal that induces upregulation of IL-1β and NLRP3, and a second signal that triggers assembly and activation of the NLRP3 inflammasome [16]. The early phase (bone marrow-derived macrophages (BMDMs) were stimulated with LPS + ATP or *Listeria monocytogenes* for not more than 1 h), acute NLRP3 inflammasome activation, depends on Toll-like receptors (TLRs) signaling via the TLR-signaling molecule IL-1 receptor-associated kinase (IRAK-1), and is independent of priming [17]. Four models for NLRP3 inflammasome activation were proposed (Figure 1). Initiation and regulation of the NLRP3 inflammasome is extensively reviewed elsewhere [18,19]. Here we focus on the roles of the ER in NLRP3 inflammasome activation.

## 3. Mitochondria-Associated ER Membranes (MAMs) Facilitate NLRP3 Inflammasome Assembly

**Figure 2:**
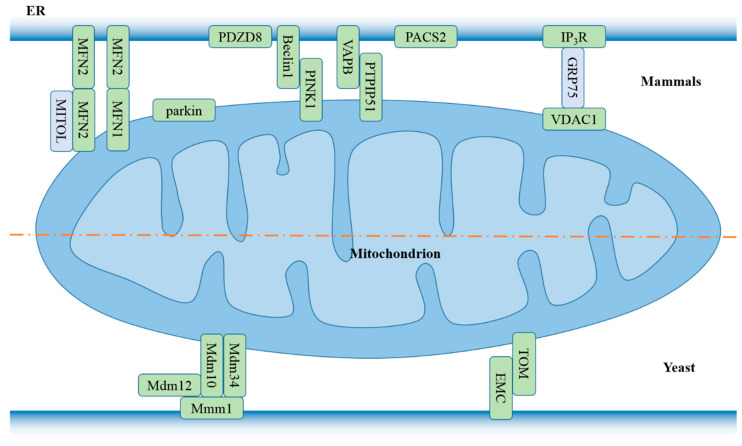
Structural components of mitochondria-associated ER membranes (MAMs). In mammalian cells, ER MFN2 tethers the ER to mitochondria by participating in homotypic or heterotypic interaction with mitochondrial MFN1 and MFN2 [44]. Mitochondrial ubiquitin ligase MITOL (also called MARCH5) interacts with and ubiquitinates mitochondrial MFN2, but not ER MFN2, to regulate MAM formation [45]. Parkin, through its N-terminal ubiquitin-like domain [46] and PDZD8 [47] engages in MAM formation, and favors Ca^2+^ transfer from the ER to mitochondria. The ER–mitochondria tethering complex, including PINK1/Beclin1 [48] and VAPB/PTPIP51 [49], enhances formation of ER–mitochondria contact sites and autophagosome. PACS2 participates in maintaining the ER–mitochondria axis [50]. In the yeast *Saccharomyces cerevisiae*, two tethering complexes are identified—Mmm1/Mdm10/Mdm12/Mdm34 complex (also known as ERMES complex) [51,52] and EMC/TOM [53]. Abbreviations: MFN2, mitofusin 2; PDZD8, PDZ domain containing 8; PINK1, PTEN induced putative kinase 1; VAPB, vesicle-associated membrane protein-associated protein B; PTPIP51, protein tyrosine phosphatase interacting protein 51; PACS2, phosphofurin acidic cluster protein 2; Mmm1, maintenance of mitochondrial morphology protein 1, Mdm10, mitochondrial distribution and morphology protein 10; ERMES, ER–mitochondria encounter structure; EMC, ER membrane protein complex; TOM, translocase of the outer membrane.

The presence of MAMs was indicated based on the electron microscope observation of continuities between mitochondria and endoplasmic reticulum in the ovaries of developing mouse and adult guinea pigs in 1969 [34]. They were first isolated from rat livers as distinct structures through cell fractionation in 1990 [35]. MAMs are specific subdomains of the ER membrane, which physically connects them to the outer mitochondria membrane and acts as a membrane contact site between mitochondria and the ER [36,37]. The distance was estimated to be ~10 nm between the smooth ER and mitochondria, and ~25 nm between the rough ER and mitochondria using electron tomography [38]. Several mitochondria- or ER-bound molecules have been reported to tether the two organelles (Figure 2). MAMs provide a platform that is crucial for calcium signaling [39], lipid homeostasis [40], autophagy [41], apoptosis [42], and tumor growth [43].

The precise location of NLRP3 inflammasome assembly is debatable. Upon stimulation, inactivated NLRP3 that resides mainly on the ER relocates to MAMs in the perinuclear space, and recruits ASC and caspase-1, triggering NLRP3 inflammasome assembly [54,55,56]. Subcellular location of resting ASC varies in different reports. It was found to be mainly in the nucleus in BMDMs [57] and carotid artery endothelial cells [58] and in the cytoplasm in THP-1 cells [56] and in BMDMs [9]. Misawa and colleagues found that ASC is located in the mitochondria, cytosol, and nucleus in BMDMs [59,60]. Caspase-1 is distributed throughout the cytoplasm and the nucleus in THP-1 cells [61]. In response to NLRP3 inducers, dynein-dependent transport of mitochondria along microtubules favors the approximation of ASC on mitochondria to NLRP3 on the ER, contributing to NLRP3 inflammasome assembly [59]. In contrast to NLRP3 inflammasome assembly in MAMs, Zhang and colleagues’ study shows that MAMs play a role in NLRP3 activation, but the assembly does not take place in the MAMs. Upon stimulation, MAMs localize near to the Golgi, and diacylglycerol (DAG) synthesis is rapidly enhanced in Golgi membranes, leading to accumulation of its effector protein kinase D (PKD) in the same organelle. Self-oligomerized NLRP3 is released from MAMs following phosphorylation at Ser293 by PKD. NLRP3 inflammasome assembly occurs in the cytoplasm. Inhibition of PKD with CRT0066101 or double knockout of PKD1-PKD3 causes retention of NLRP3 in MAMs close to the Golgi, preventing ASC recruitment to NLRP3 and inflammasome activation. [62,63].

NLRP3 oligomerization, at least trimerization but not dimerization, is necessary for its activation [64], but whether resting NLRP3 on the ER is in a monomeric or oligomeric state remains elusive. NLRP3 and NLR family CARD domain-containing protein 4 (NLRC4) share the NACHT domain and LRR domain. The two domains are distant from each other and are coupled through a β-hairpin. LRR domain inhibits NLRC4 self-oligomerization through its NACHT domain, sequestering the protein in a monomeric and inactive state. LRR deletion results in a constitutively-active NLRC4 [65]. Similarly, both insect cell- and *Escherichia coli*-expressed NLRP3^PYD-NBD^ induces increased ASC^PYD^ polymerization, while the highly-aggregated NLRP3^FL^ shows less activity [66]. Under confocal microscopy, NLRP3 localizes diffusedly throughout cytoplasm in resting cells, and NLRP3 foci are observed in response to agonists [67,68,69]. Hence, it is believed that NLRP3 exists in a monomeric state in unstimulated cells, and NLRP3 agonists induces its oligomerization [14]. However, to our knowledge, no direct evidence demonstrates this. Both NLRP3^PYD^ monomers and high order oligomers exist in solution, and the presence of salt favors NLRP3^PYD^ self-association [70]. Compan and colleagues’ study showed that PYD of one NLRP3 is in spatial proximity to the LRR domain of adjacent NLRP3 in the resting state, which leads to formation of NLRP3 complexes on the ER. Treatment with NLRP3 stimuli induces conformational change of NLRP3 without dissociation of the complexes [56,71]. 

**Figure 3:**
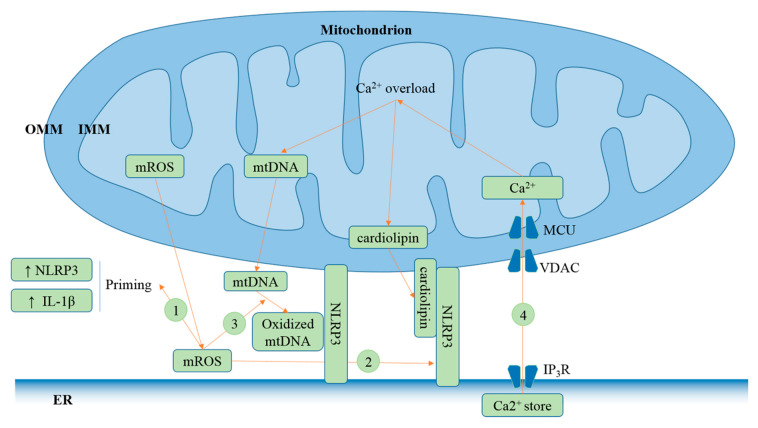
MAMs contribute to NLRP3 inflammasome activation. (1) ER–mitochondria proximity plays a crucial role in the signaling pathways associated with short-lived ROS. Its generation induces increased expression of NLRP3 and substrate of the inflammasome IL-1β [30]. NLRP3 level is critical for the priming step. The inflammasome can be activated by ATP alone without lipopolysaccharide (LPS) priming in macrophages overexpressing NLRP3 [74]. (2) mROS promote NLRP3 association with mitochondria [73], which subsequently induces its interaction with cardiolipin that translocates from the inner mitochondrial membrane (IMM) to the outer mitochondrial membrane (OMM) in response to the NLRP3 stimuli [72]. (3) mROS mediate oxidation of mtDNA [75]. Oxidized mtDNA binds to NLRP3, triggering NLRP3 inflammasome activation [7]. (4) The close proximity between the ER and mitochondria facilitates rapid Ca^2+^ mobilization to mitochondria from the ER through mitochondrial calcium uniporter (MCU) and voltage-dependent anion-selective channel (VDAC), leading to mitochondrial Ca^2+^ overload, mitochondrial dysfunction, release of mtDNA and cardiolipin externalization [31,33,76].

MAMs constitute a signaling hub regulating NLRP3 inflammasome activation (Figure 3). The tight spatial relationship between the ER and mitochondria facilitates NLRP3 association with oxidized mitochondrial DNA (mtDNA) [7], cardiolipin that translocates to the OMM [72], and rapid Ca^2+^ influx into mitochondria from ER stores [31], triggering NLRP3 inflammasome activation. Of the four models mentioned above, Ca^2+^ mobilization and K^+^ efflux triggering NLRP3 inflammasome activation are well supported. Meanwhile, Ca^2+^ mobilization functions upstream of K^+^ efflux [20]. ROS are short lived and act as a signaling messenger for a short distance. Thus, ER–mitochondria proximity contributes to its roles in priming [30], NLRP3 localization to mitochondria [73], and mtDNA oxidation which is associated with NLRP3 inflammasome activation [7].

## 4. ER Stress Participates in NLRP3 Inflammasome Activation

**Figure 4:**
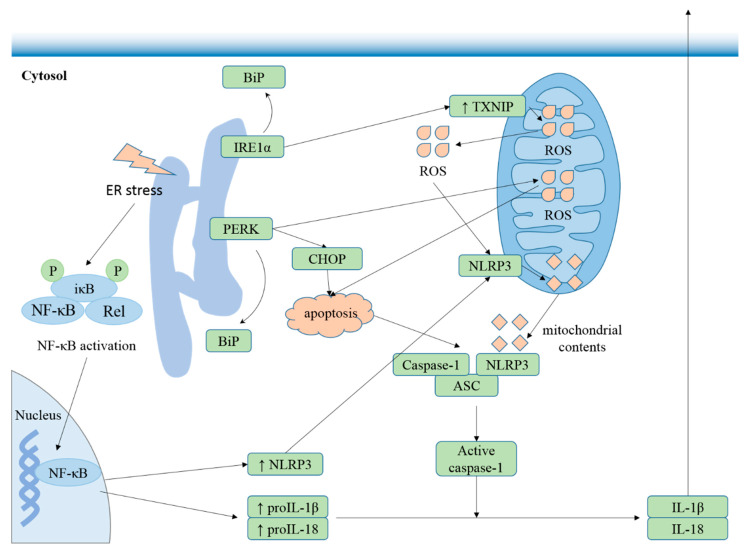
ER stress is involved in NLRP3 inflammasome activation. ER stress promotes expression of IL-1β and NLRP3 via the NF-κB pathway, contributing to NLRP3 inflammasome activation [79]. In response to ER stress, IRE1α reversibly dissociates from the endoplasmic reticulum chaperone immunoglobulin-binding protein (BiP) [80], induces thioredoxin interacting protein (TXNIP) translocation to mitochondria, ROS release, and NLRP3 association with mitochondria, promoting release or externalization of mitochondrial contents, which bind and activate NLRP3, provoking secretion of proinflammatory cytokines such as IL-1β and IL-18 [73]. Protein kinase-(PKR)-like eukaryotic initiation factor 2α kinase (PERK) contributes to apoptosis by sustaining the level of C/EBP-homologous protein (CHOP) and facilitating the propagation of ROS signals after dissociation from BiP [81,82,83], and apoptosis acts as the second signal to activate NLRP3 [7].

Recent studies suggest that ER stress is involved in inflammation [77]. In eukaryotic cells, three ER-stress sensors, endoribonuclease inositol-requiring enzyme 1 (IRE1), double-stranded RNA-activated protein kinase-(PKR)-like eukaryotic initiation factor 2α kinase (PERK), and the activating transcription factor-6 (ATF6), provoke an adaptive program, defining the fate of the stress cells following sensing unfolded proteins [78]. IRE1α and PERK induce NLRP3 inflammasome activation via the NF-κB pathway (Figure 4), while few reports show that ATF6 couples ER stress to the NLRP3 inflammasome.

### 4.1. ER Stress

The ER is a large and dynamic endomembrane compartment that forms an interconnected network possessing two major domains with various structures and functions. The two major domains of the ER are the nuclear envelope which is made up of a single flat ER membrane bilayer, and the peripheral ER whose branches expand from the outer nuclear membranes into the cytosol and generate an interconnected network of flat cisternal sheets and reticulated tubules [11]. ER sheets are the key regions for protein translation, folding, translocation, assembly of newly biosynthesized secretory proteins, and posttranslational modification, while ER tubules may be the key locations for lipid synthesis and signal transducing between the ER and other organelles [11,84]. Nascent secretory and membrane proteins translocate into the ER lumen, and start to fold co-translationally. Both co-translation and post-translation need specific and sequential interaction with chaperone. Perturbations, including changes in calcium homeostasis, infection, and hypoxia, lead to generation of misfolded and/or unfolded proteins. The accumulation of misfolded and/or unfolded proteins exceeds the capacity of the ER to process newly-biosynthesized proteins, and the ER fails to cope with the excess protein load, which is termed ER stress. An integrated intracellular signaling cascade is triggered to avert ER stress called the unfolded protein response (UPR) [12]. During ER stress, synthesis of folding catalysts and ER-resident chaperones is increased to promote the folding capacity. Protein translation is attenuated and degradation of misfolded proteins is induced to decrease the folding load in the ER [85].

### 4.2. IRE1 and the NLRP3 Inflammasome

IRE1, a bifunctional type I transmembrane kinase and endoribonuclease, is the most evolutionarily-conserved UPR transducer [86]. It was originally identified to be required for inositol phototrophy in *Saccharomyces cerevisiae* because IRE1 mutation caused myoinositol auxotrophy [87]. Two homologs, referred to as IRE1α and IRE1β, exist in both the murine and human genomes [88]. IRE1α is ubiquitously expressed in all cell types and has been extensively described [89]. It exerts a stronger X-box binding protein 1 (XBP1) mRNA splicing activity [90]. IRE1β is expressed in epithelial cells of the gastrointestinal tract [91] and human bronchial epithelia [92]. It has a stronger activity of regulated IRE1-dependent decay of mRNA (RIDD) [90]. 

IRE1α is highly conserved from yeast to humans. IRE1α is composed of an N-terminal ER-luminal domain, a single-pass transmembrane segment and a C-terminal cytoplasmic domain encompassing a Ser/Thr protein kinase domain and an endoribonuclease (RNase) domain [93]. Ire1 kinase domain in the cytoplasm or nucleus is trans-autophosphorylated mutually on yeast Ser840, Ser841 [94], and Thr844 [95] as a result of oligomerization, and transmits unfolded protein signal across the membrane. A 133-amino-acid globular RNase domain that locates after the kinase domain confers endonuclease activity to IRE1α, and induces independent cleavage of the 5′ and 3′ splice junction of *Hac1* mRNA in yeast [96] and *XBP1* mRNA in metazoans [97]. 

IRE1α forms heterodimers with BiP at a 1:1 molar ratio, and subsequently generates a stable complex of relative molecular mass 140–230 kDa in unstressed pancreatic acinar cell line AR42J [83]. During ER stress, BiP reversibly dissociates from the heterodimers in the complex, initiating signaling transduction in the unfolded-protein response. Centrally-located residues of IRE1α cLD (conserved core region of the luminal domain) link two neighboring IRE1α monomers, which initiates IRE1α homodimerization. Further interaction between cLD dimers forms higher-order oligomers (called clusters in Kimata et al.’s study, that display a dot-like distribution [98]) [99], as well as a complex of higher relative molecular mass [83]. Local concentration of the kinase domains causes transphosphorylation of tyrosine residues [100], evoking a conformational change that promotes Ire1 RNase activity [99]. Then, the deep groove in IRE1α cLD captures and interacts directly with the unfolded proteins, which inhibits their aggregation and initiates UPR [98]. Additionally, Walter’s studies show that yeast Ire1 cLD directly binds to unfolded proteins containing basic and hydrophobic residues and then oligomerizes [101], and mutated Ire1 that lacks the BiP binding site still properly responds to ER stress [102].

In mouse embryo fibroblasts (MEFs), activated IRE1α provokes upregulation of TXNIP mRNA and protein by decreasing levels of miR17, a TXNIP-destabilizing micro-RNA, following stimulation with ER-stress agents such as tunicamycin that inhibits N-linked glycosylation or thapsigargin that inhibits the sarcoplasmic-endoplasmic reticulum calcium ATPase pump. Inhibition of IRE1α RNase with STF-083010 prevents thapsigargin-induced TXNIP mRNA expression [103]. TXNIP induction is dependent on IRE1α’s RNase catalytic activity and independent of XBP1. Trans-autophosphorylation of IRE1α RNase domain potentiates its RNase, but is dispensable for TXNIP induction [103]. 

ER chaperone BiP is associated with mitigation of ER stress. BiP overexpression in Chinese hamster ovary (CHO) cells blocks secretion of human factor VIII [104], and attenuates inducibility of CHOP [105] whose expression increases in response to ER stress [106]. Mutation in the BiP substrate-binding domain disrupts the association of BiP to Ire1α and elicits complete cleavage of Hac1 even in the absence of extrinsic ER stress. BiP fails to dissociate from Ire1, and cleavage of Hac1 is inhibited in the presence of tunicamycin in yeast cells harboring a mutation in the ATPase domain [80]. 

IRE1α is a crucial regulator of NLRP3 inflammasome activation triggered by ER stress [107], and also a potential target for inflammation-associated diseases, including arthritis [108], sepsis [109], atherosclerosis [110], and viral myocarditis [111]. IRE1α controls cell fate through its RNase outputs: XBP1 mRNA splicing that has a prosurvival output and RIDD that has a proapoptotic output [112]. High-fat diet (HFD)-fed Ern1^−/−^ mice exhibit decreased serum levels of tumor necrosis factor (TNF), IL-1β and the chemokine monocyte chemotactic protein-1 (MCP-1, also called C–C motif chemokine ligand 2, CCL2) [113]. Following infection with *Brucella abortus* vaccine strain RB51, mice treated with 4μ8C which selectively inhibits IRE1α RNase activity [114] show less serum IL-1β and increased susceptibility to RB51, as reflected by increased bacterial burden in the spleen [73].

Under ER stress, activated IRE1α contributes to increased expression of TXNIP through induction of miR17 degradation. TXNIP shuttles to the mitochondria and associates with thioredoxin-2 [103], favoring release of ROS from mitochondria. Due to the increased concentration of ROS, NLRP3 which resides mainly on the ER redistributes to mitochondria, triggering caspase-2 cleavage and recruitment to mitochondria. Of note, ASC or caspase-1 is dispensable for caspase-2 recruitment. Then, NLRP3 and caspase-2 mitochondrial recruitment causes cardiolipin-dependent truncation and activation of BH3 interacting domain death agonist (BID) [73,115]. BID, a pro-apoptotic factor of the Bcl-2 family, which acts as a membrane-targeted and concentrated-death ligand, oligomerizes BAK or Bax into pores that elicit the release of mitochondrial contents such as cytochrome c [116]. Activated BID leads to exposure of the N-terminal domain of Bax through its conformational change, Bax oligomerization and the Bax integration into the OMM through the direct interaction between Bax and BID [117,118], openings of supramolecular size in the OMM [119,120], and subsequent release of cytochrome c and other mitochondrial contents, including cardiolipin [121] and mitochondrial DNA (mtDNA) [122]. Cardiolipin [72] or oxidized mtDNA [7] directly binds and activates NLRP3, provoking NLRP3 inflammasome assembly and resultant caspase-1 maturation as well as release of proinflammatory cytokines, including IL-1β and IL-18. Tunicamycin- or RB51-induced release of mtDNA is blocked by NLRP3 deletion, or IRE1α inhibition with 4μ8C or specific siRNA, but not inhibition of ASC or caspase-1 [73]. However, Menu and colleagues’ study showed that secretion of mature IL-1β is not affected in IRE1α-silenced THP-1 cells following tunicamycin stimulation [123].

The role of TXNIP in regulation of NLRP3 inflammasome signaling by IRE1α was also demonstrated in other reports. TXNIP silencing using siRNA inhibits NLRP3 inflammasome activation in response to ethanol [124], trimethylamine-N-oxide [125], fructose [126], or simulated ischemia/reperfusion injury [127]. In contrast, Bax is not necessary in some circumstances. Caspase-1 and IL-1β activation is not prevented in LPS-primed BMDMs isolated from Bak^−/−^Bax^−/−^ mice following stimulation with nigericin, staurosporine, or ultraviolet irradiation [128,129]. Whether IRE1α regulates NLRP3 inflammasome activation induced by LPS + ATP is controversial. Zhang and colleagues found that treatment with LPS + ATP fails to cause ER stress in BMDMs, as reflected by the absence of the spliced XBP-1 mRNA [130]. Inhibition of IRE1α with 4μ8C or tauroursodeoxycholic acid (TUDCA) which alleviates ER stress, or silencing of Ern1, has no effect on LPS + ATP-induced caspase-1 maturation and IL-1β secretion in BMDMs [73]. However, Talty and colleagues’ study show that stimulation with LPS + ATP leads to splicing of XBP1 mRNA in human peripheral blood mononuclear cells (PBMCs) [107], and Chop deletion reduces the caspase-1 maturation and IL-1β secretion in BMDMs [32]. Treatment of BMDMs with 4μ8C impedes NLRP3 inflammasome activation triggered by LPS + ATP [110].

It seems that TLR2 and TLR4 link IRE1α to the NLRP3 inflammasome. Priming of the NLRP3 inflammasome involves upregulation of key components, including NLRP3 and IL-1β through TLR2 or TLR4 [17,30]. Meanwhile, TLR2 or TLR4 triggers IRE1α activation and subsequent XBP1 splicing, which are dependent on TNF-receptor-associated factor 6 (TRAF6) and NADPH oxidase 2 (NOX2). The TLR4 ligand LPS provokes IRE1α-dependent NLRP3 inflammasome activation in the presence of nigericin. Treatment with the TLR3 agonist poly(I:C), TLR7 agonist imiquimod, or TLR9 agonist ODN2006, fails to trigger XBP1 activation [107]. However, TLR4 is not always involved in IRE1α-dependent NLRP3 inflammasome activation. IRE1α inhibition does not lead to decreased caspase-1 cleavage and IL-1β production induced by LPS + ATP. In addition, LPS is able to induce XBP1 splicing [107], but IL-1β transcription is not affected by XBP1 deficiency following treatment with LPS without signal II [131]. 

### 4.3. PERK and the NLRP3 Inflammasome

PERK is also a type I transmembrane protein kinase that transmits stress signals in response to perturbations in ER protein [132]. PERK–BiP heterodimers form a complex of relative molecular mass approximately 230 kDa in untreated AR42J cells. Treatment with thapsigargin, tunicamycin, or dithiothreitol (DTT) results in reversible BiP dissociation from the complex, formation of a PERK-containing complex of higher relative molecular mass (more than 600 kDa) and PERK transphosphorylation. BiP reassociates with PERK and PERK is converted from a phosphorylated form to a dephosphorylated form after removal of DTT [83,133]. The N-terminal luminal domain sequences of PERK, which sense the accumulation of unfolded proteins in the ER, align well with the corresponding region of IRE1α-cLD [99]. Oligomerization of the N-terminal domain favors PERK transphosphorylation of the C-terminal cytoplasmic kinase domain at Thr980 on the kinase activation loop [134]. Activated PERK potentiates phosphorylation of eukaryotic initiation factor 2α (eIF2α) on Ser51 through its kinase domain [134]. Phosphorylation of eIF-2α impairs the exchange of guanosine 5′-triphosphate (GTP) for guanosine diphosphate (GDP) on eIF-2α by competitively binding eIF-2β, and interferes with the formation of a 43S preinitiation complex, which leads to decreased rates of initiation of protein translation and decreases the load of proteins entering the ER [132].

PERK is linked to the NLRP3 inflammasome by apoptosis and MAMs. ROS-mediated ER stress relies on PERK to propagate apoptosis [81], and apoptosis is believed to function as the second signal for NLRP3 inflammasome activation [7]. PERK is a component of the MAMs. Loss of PERK leads to a fragmented ER morphology and curtails the ER–mitochondria connections via its cytoplasmic domain, which reduces the propagation of ROS signals to the neighboring mitochondria, and attenuates intrinsic apoptosis induced by ROS-based ER stress. Meanwhile, PERK inhibition disturbs Ca^2+^ release from ER stores due to the perturbation of the MAMs, further impeding NLRP3 inflammasome activation [90,135]. Studies also show that PERK may engage in NLRP3 inflammasome activation. PERK silencing leads to decreased protein expression of NLRP3 in the tunicamycin-treated hepatocyte-derived AML12 cell line [136]. Curcumin [137] or ilexgenin A [138] plays an inhibitory in PERK and IRE1α phosphorylation, apoptosis, and NLRP3 inflammasome activation. However, NLRP3 inflammasome activation is not always dependent on PERK. Tunicamycin treatment triggers apoptosis through ROS generation [139] and IL-1β cleavage which is dependent on NLRP3 inflammasome activation [123]. Additionally, tunicamycin induces PERK-dependent upregulation of TXNIP mRNA and protein [103], but PERK silencing shows no change in the secretion of mature IL-1β [123].

### 4.4. ATF6 and the NLRP3 Inflammasome

ATF6 is a type II transmembrane glycoprotein with a hydrophobic stretch anchoring in the ER membrane. It is constitutively expressed as an inactive 90 kD protein [140]. During ER stress, Ca^2+^ release from ER stores leads to the fragmentation of Golgi membranes, and ATF6 redistributes to the Golgi after dissociation from BiP. ATF6 accumulation disrupts the ER/Golgi membrane network [141,142]. Meanwhile, Site-1 protease (S1P) and Site-2 protease (S2P) that are synthesized in the ER as inactive precursors translocate to the Golgi, become active after autocatalytic cleavage [143,144], and process ATF6 into its active form, a soluble 50 kD protein. Active ATF6 moves to the nucleus and activates transcription of genes encoding chaperones that restore protein folding in the ER lumen [140,145]. Most reports indicate that ATF6 does not contribute to NLRP3 inflammasome activation. ATF6 inactivation has no effect on TXNIP levels and the NLRP3 inflammasome [103,123,136]. ATF6 expression remains unchanged following stimulation with LPS [82,131]. However, cleaved ATF6 plays a critical role in pyroptosis and NLRP3 inflammasome activation induced by silver nanoparticles. Inhibition of ATF-6 cleavage with the S2P inhibitor 1,10-phenanthroline blocks caspase-1 maturation, IL-1β secretion, and pyroptosis [146]. 

## 5. ER Ca^2+^ Signaling Contributes to NLRP3 Inflammasome Activation

Ca^2+^ signaling is a universal and versatile mechanism that engages in a wide range of fundamental cellular events, including autophagy [147], apoptosis [148], inflammasome [76], differentiation [149], proliferation [150], secretion [151], and gene expression [152]. Signaling precision needs localized increases in the cytosolic Ca^2+^ level due to presence of plenty of potential cellular targets throughout the cell. The ER, the major intracellular Ca^2+^-storage organelle, plays a critical role in Ca^2+^ signaling. Although most of the Ca^2+^ in the ER compartment is bound to Ca^2+^- binding proteins such as calreticulin or calsequestrin, free Ca^2+^ level is rigorously controlled [153,154]. The ER is physically and functionally linked to other organelles such as mitochondria and lysosomes, establishing close contact sites and possessing a rapid effect on their physiological function [155]. The ER functions as a sink for Ca^2+^ that enters cells via the channels, and also a store for Ca^2+^ that is released into the cytosol [156]. It is generally accepted that the ER has one continuous Ca^2+^ store, but emerging evidence reveals several apparently discrete ER Ca^2+^ stores [157].

Excessive and/or sustained Ca^2+^ mobilization from the ER to mitochondria results in mitochondrial Ca^2+^ overload, triggering NLRP3 inflammasome activation [32]. Upon stimulation with calcium-sensing receptor (CASR) agonists, CASR stimulates the activity of phospholipase C (PLC), leading to phosphatidylinositol-4,5-bisphosphate (PIP2) hydrolysis into DAG. DAG induces inositol trisphosphate (InsP3) interaction with InsP3 receptors (InsP3Rs), resulting in Ca^2+^ release from ER stores [33]. Ca^2+^ influx into mitochondria through mitochondrial calcium uniporter (MCU) in the IMM and voltage-dependent anion-selective channel (VDAC) in the OMM causes Ca^2+^ overload and resultant mitochondrial destabilization [32], leading to release or externalization of mitochondria-derived molecules such as mtDNA [7] and cardiolipin [72]. Oxidized mtDNA and cardiolipin directly bind and activate NLRP3, and consequently cause maturation of caspase-1, pyroptosis and secretion of proinflammatory cytokines.

## 6. Lipid Perturbation and Cholesterol Trafficking to the ER Activate the NLRP3 Inflammasome

The ER is the main site of lipid synthesis [158]. Cellular lipids are major components of cell membranes and signaling messengers. Cholesterol is a vital lipid that carries out diverse functions, including host defense [159] and maintenance of membrane integrity and fluidity [160]. Levels of intracellular cholesterol are maintained through de novo biosynthesis in the ER and uptake of low-density lipoproteins (LDLs) [161]. Cellular cholesterol content is controlled via a feedback mechanism [162]. In states of low ER cholesterol, the escort protein sterol response element-binding protein (SREBP) cleavage-activating protein (SCAP) dissociates from the insulin-induced gene (INSIG) and binds to SREBP2. The SCAP–SREBP2 complex translocates to the Golgi, where SREBP2 is cleaved by S1P and S2P. The cleaved SREBP2 enters the nucleus and activates the transcription of genes that participate in cholesterol biosynthesis and uptake. Increased cholesterol content results in SCAP–INSIG association and SREBP2 sequestration in the ER [163].

Lipids such as saturated fatty acids induce ER stress in both yeast [164] and mammalian cells [165]. Lipid perturbation activates UPR signaling independently of the influence on protein folding in the ER lumen [166]. The ER-spanning transmembrane domain of IRE1α and PERK, rather than the luminal ER-stress-sensing domain, is indispensable for their activation in response to increased lipid saturation [167]. Silencing of mdt-15, a subunit of Mediator that is critical for expression of genes participating in fatty acid metabolism, the lipid metabolism enzymes stearoyl-CoA-desaturases (SCD), or S-adenosyl methionine synthetase (sams-1), activates the UPR, but does not promote misfolded protein aggregates [168]. Inhibition of SCAP–SREBP2 ER-to-Golgi translocation with betulin, or SCAP silencing, suppresses caspase-1 maturation and IL-1β secretion induced by ATP, nigericin, MSU, and alum in LPS-primed macrophages [169]. Lipid perturbation-induced UPR can be bridged to the NLRP3 inflammasome by Ca^2+^ signaling. Increased phosphatidylcholine/phosphatidylethanolamine ratio in the ER leads to decreased Ca^2+^ transport activity through sarco/endoplasmic reticulum calcium ATPase (SERCA) [158]. Thapsigargin inhibits the Ca^2+^ transport activity of SERCA, induces a rapid Ca^2+^ leak from Ca^2+^ stores and thereby increases the concentration of cytosolic free Ca^2+^ [170,171], triggering NLRP3 inflammasome activation [123].

Trafficking of cholesterol to the ER triggers NLRP3 inflammasome activation. Deficiency in lysosomal cholesterol transporter Niemann–Pick C1 (NPC1) through which cholesterol translocates to other cellular sites including the ER, or inhibition of cholesterol trafficking with itraconazole, U18666a or stains, results in reduced caspase-1 activation and IL-1β secretion. Trafficking of cholesterol to the ER is dispensable for AIM2 inflammasome activation [161]. Knockout of cholesterol-25-hydroxylase that produces 25-hydroxycholesterol from cholesterol enhances caspase-1 maturation [172] and IL-1β secretion [173] following treatment with LPS + ATP. SREBP2 and SCAP that are involved in cholesterol trafficking are required for optimal NLRP3 inflammasome activation. Overexpression of mature form of SREBP2 contributes to NLRP3 inflammasome activation after exposure to atheroprone oscillatory shear flow, while silencing SREBP2 using siRNAs reduces cleavage of caspase-1 and IL-1β in endothelial cells [174]. Increased extracellular cholesterol content does not trigger NLRP3 inflammasome activation. Treatment with cholesterol that is solubilized by forming complexes with methyl-β-cyclodextrin (MCD) provokes IL-1β secretion in an AIM2 inflammasome-, but not NLRP3 inflammasome-dependent manner in LPS-primed BMDMs [173]. However, the reason why the NLRP3 inflammasome is not activated still needs to be explored. LPS + cholesterol/MCD induces increased ROS production and release of mtDNA [173], which is supposed to activate the NLRP3 inflammasome [7].

## 7. Induction of ER Stress Causes NLRP3-Dependent Release of Cytokines

ER stress induces NLRP3 inflammasome activation, regulating release of cytokines. Treatment with TUDCA, or 4μ8C, or silencing Ern1 attenuates RB51-induced IL-1β release. 4μ8C-treated mice display decreased serum IL-1β levels [73]. Silver nanoparticles induce IL-1β secretion which is dependent on NLRP3 and ATF-6 cleavage [146]. Ilexgenin A inhibits palmitate-induced ER stress by attenuating phosphorylation of PERK and IRE1α, decreases IL-1β release, and enhances IL-6 secretion [138]. Treatment with metformin, resveratrol, or TUDCA impedes eIF2α and IRE1α phosphorylation, inhibiting IL-6 and MCP-1 production induced by high glucose [175]. Pretreatment with curcumin inhibits PERK and IRE1α activation, and prevents IL-1β release induced by oxygen- and glucose-deprivation in the hippocampus [137]. TUDCA treatment restrains upregulation of IL-1β, IL-18, and NLRP3 induced by LPS, and also decreases the serum levels of IL-1β, TNFα, interferon γ (IFN-γ), MCP-1, and IL-6 in LPS-treated mice [82].

IL-1β, a potent proinflammatory cytokine, is the best-described IL-1 family member that is essential for host defense against infection and injury [176]. IL-18, another IL-1 family member, also formerly called IFN-γ–inducing factor, plays a critical role in Th1 response through inducing IFN-γ production in T cells and natural killer cells. Both IL-1β and IL-18 are synthesized as biologically-inactive precursors which lack signal peptides, and are cleaved into mature molecules by caspase-1 [177]. IL-6 exerts its anti-inflammatory effect via the membrane bound IL-6 receptor (IL-6R), and proinflammatory effect via the soluble IL-6 receptor in body fluids such as urine and blood [178]. MCP-1, a potent chemokine, recruits monocytes into foci of inflammation [179]. TNFα secretion occurs independently of NLRP3 inflammasome activation [7]. Its generation at high concentration results in the development of inflammatory responses [180]. IFN-γ, an essential endogenous regulator of immune response, is involved in antiviral and antimicrobial defense [181] as well as immune modulation in cancer [182].

## 8. Concluding Remarks

The ER is the major site for protein folding and trafficking and is critical to many cellular functions. In response to the agonists, NLRP3 localizes to the MAMs and forms active self-oligomerized conformation in the MAMs. Whether NLRP3 inflammasome assembly takes place in the MAMs or cytoplasm still needs to be further investigated. The close proximity between the ER and mitochondria in the MAMs facilitates the interaction between NLRP3 and its ligands that are released from or reside on mitochondria, and rapid Ca^2+^ influx into mitochondria from ER stores. Failure of the ER’s adaptive capacity promotes NLRP3 inflammasome activation. Following RB51 infection, IRE1α activation increased ROS production, promoting recruitment of NLRP3 and caspase-2 to the mitochondria, leading to release of mitochondrial contents and subsequent NLRP3 inflammasome activation. Lipid perturbation and trafficking of cholesterol to the ER triggers NLRP3 inflammasome activation. ATF6 cleavage is required for silver nanoparticle-induced NLRP3 inflammasome activation. Exploration of the roles of the ER in NLRP3 inflammasome activation contributes to a better understanding of innate immunity and provides potential therapeutic targets for treating inflammatory diseases.
